# Relationship between pattern electroretinogram and optic disc morphology in glaucoma

**DOI:** 10.1371/journal.pone.0220992

**Published:** 2019-11-07

**Authors:** Soo Ji Jeon, Hae-Young Lopilly Park, Kyoung In Jung, Chan Kee Park

**Affiliations:** Department of Ophthalmology, Seoul St. Mary’s Hospital, College of Medicine, The Catholic University of Korea, Seoul, Republic of Korea; National Taiwan University Hospital, TAIWAN

## Abstract

**Purpose:**

To evaluate the relationship between pattern electroretinogram (PERG) and optic disc morphology in glaucoma suspect and glaucoma.

**Methods:**

Eighty-six eyes of glaucoma suspect and 145 eyes of manifest glaucoma subjects were included in this study. Average peripapillary retinal nerve fiber layer (RNFL) thickness was obtained with spectral-domain optical coherence tomography, and optic disc imaging was performed using the Heidelberg Retinal Tomograph (HRT). Visual function was evaluated with perimetry (SITA and frequency doubling technology) and PERG. Scatter plots and correlation coefficients were evaluated between visual function and RNFL thickness or optic disc structure.

**Results:**

Scatter plots of PERG and perimetry according to RNFL thickness change showed that PERG started to decrease earlier than did perimetry. The differences between linear and logarithmic R^2^ were largest for the scatter plot of SITA 24–2 (linear R^2^ = 0.415; logarithmic R^2^ = 0.443) and the smallest for P50 amplitude of PERG (linear R^2^ = 0.136, logarithmic R^2^ = 0.138). In glaucoma suspect, HRT parameters such as cup shape measure (CSM) and linear cup-disc ratio (CDR) had significant correlations with PERG amplitudes (*P =* 0.016 for P50 and 0.049 for N95 in CSM, *P =* 0.012 for P50 in CDR). However, in glaucoma patients, mean RNFL thickness was associated with PERG amplitude (*P =* 0.011 for P50 and 0.002 for N95).

**Conclusions:**

PERG deterioration occurred earlier than did perimetry according to RNFL thickness decrease. PERG amplitudes were significantly correlated with disc morphology in glaucoma suspect. These results suggest that PERG can detect ganglion cell dysfunction before the cells die.

## Introduction

Glaucoma is diagnosed with morphological changes to the optic disc, thinning of the ganglion cell layer, and defects in corresponding areas in the visual field. In early glaucoma, however, there have been difficulties in identifying glaucomatous damage because of the discrepancy between the timings of structural and functional losses. It is suggested that structural thinning of the nerve fiber layer precedes defects in visual function and that retinal ganglion cell (RGC) redundancy could account for this characteristic phenomenon.[1–3] The differences in decibel scale in perimetry and linear scale in structural parameters also makes it difficult to match the amount of axonal thinning with visual functional damage.[4] Curvilinear relationships between the structural and functional tests in early glaucoma patients lead to the status of “preperimetric glaucoma,” and our team has reported multiple attempts to overcome these deficiencies in standard perimetry.[3,5,6]

In recent decades, a number of researchers have attempted to investigate RGC function directly using electrophysiological tests.[7–11] Perimetry is an indirect method of testing visual function because it records the patient’s response after the visual cortex has recognized the light. However, pattern-evoked electroretinogram (PERG) is an objective method of measuring RGC function, and the noteworthy feature of PERG is that it could detect dysfunctional RGC without structural axonal loss.[12] Salgarello et al.[10] reported that even subjects with ocular hypertension and normal visual field test had PERG results changes that were related to optic disc cup morphology. In other studies, authors reported PERG changes after acute intraocular pressure (IOP) modification before histological loss of RGC.[13,14] In consideration of these findings, the temporal sequences of structural damage and functional change might be rearranged using electrophysiological tests. If dysfunctional RGC status with maintained cell structure exists prior to substantial loss of RGC, the early retinal neuron dysfunction detected by electrophysiological testing may be meaningful in expecting further progression of disease.

As mentioned above, glaucomatous changes have been known to occur following the sequence of enlarging cup-disc (C/D) ratio, thinning of retinal nerve fiber layer (RNFL), and defect of visual field (VF) test. The aim of this study was to investigate the changes in RGC function before structural changes such as RNFL thickness decrease and the possibility of overcoming late VF deterioration, especially in glaucoma suspect using PERG. Furthermore, we hypothesized that if there is a diverse range of RGC function even in glaucoma suspects, patients with dysfunctional RGC may have earlier changes in the PERG and there could be PERG-related ocular parameters in glaucoma suspects. We may obtain clinical ocular parameters to identify glaucoma suspects with early functional changes.

## Materials and methods

### Subjects

We performed this cross-sectional study according to the tenets of the Declaration of Helsinki and was approved by the Institutional Review and Ethics Boards of Seoul St. Mary’s Hospital, South Korea. 86 eyes from 54 patients of glaucoma suspect and 145 eyes from 84 normal-tension glaucoma (NTG) patients from the glaucoma clinic of Seoul St. Mary’s Hospital between September 2017 and February 2018 were included. The need for written informed consent was waived by our Review Board.

Each subject underwent comprehensive ophthalmic examinations including slit-lamp examination, Goldmann applanation tonometry, gonioscopy, and dilated fundus bimicroscopy. Through stereoscopic optic disc photography, two glaucoma specialists (SJJ and HYP) independently evaluated vertical C/D ratio. We only included in the study subjects for whom the two observers agreed on their C/D ratios.

All subjects underwent standard automated perimetry (SAP) using SITA program (Humphrey Visual Field Analyzer; Carl Zeiss Meditec, Inc, Dublin, CA, USA) and frequency doubling technology (FDT) perimetry (Carl Zeiss). Circumpapillary RNFL thickness was measured using Cirrus spectral-domain optical coherence tomography (OCT; Carl Zeiss).

Inclusion criteria for this study were as follows: (1) IOP ≤ 21 mmHg with or without IOP-lowering medication, (2) best corrected visual acuity of 20/40 or better, (3) open angle on gonioscopy, (4) spherical equivalent within ± 5.0 diopters, and (5) no history of disease affecting visual pathways, retinal disease, optic neuritis, or presence of diabetes.

All NTG patients had glaucomatous optic discs (increased C/D ratio with localized loss or thinning of neuroretinal rim, generalized loss of disc rim, or peripapillary disc hemorrhage) and VF defects. Glaucomatous VF defects satisfied following the conditions: glaucoma hemifield test results were outside normal limits at either ≥ 3 adjacent points with p < 0.05 or ≥ 2 adjacent points with p < 0.02 on a pattern deviation probability map. We considered the VF results reliable when fixation loss was < 20%, the false-positive rate was < 15%, and the false-negative rate was < 15%.

We defined glaucoma suspect as not having glaucomatous VF defects on both SAP and FDT and normal RNFL thickness in OCT results with only vertical C/D ratio ≥ 0.5 or asymmetric optic discs in both eyes (asymmetry of C/D ratio between two eyes more than 0.2 that was not caused by the difference in optic disc size or shape). We considered RNFL thickness to be normal if it was within 95% of the internally embedded database of healthy, age-matched normal population and it was marked in the green color sector of the temporal-superior-nasal-inferior-temporal (TSNIT) graph.

### PERG examinations

The electrophysiologic test results were recorded using a PERG system (Neuro-ERG, Neurosoft, Ivanovo, Russia) by one trained examiner. The subjects were seated in front of a display in a semi-dark room that had constant background illumination of 50 lux and had full optical correction according to own refraction before examination. 35 mm Ag/AgCl ground electrodes were placed on the earlobes, and the same type of active electrodes were placed on the skin near the contours of the lower eyelid on the ipsilateral side. Both eyes were examined simultaneously. The visual stimulus was a checkerboard pattern with mean luminance of 300 cd/m^2^ and contrast between black and white squares of 98%. The patterns on display were reversed at the rate of 4 reversals per second at a 60-cm distance from the patients. The stimulus monitor screen covered 48° of the visual field with each check size of 1.8° visual angle. All subjects were instructed to focus intensely on the red fixation target at the center of the monitor screen.

**Fig 1:**
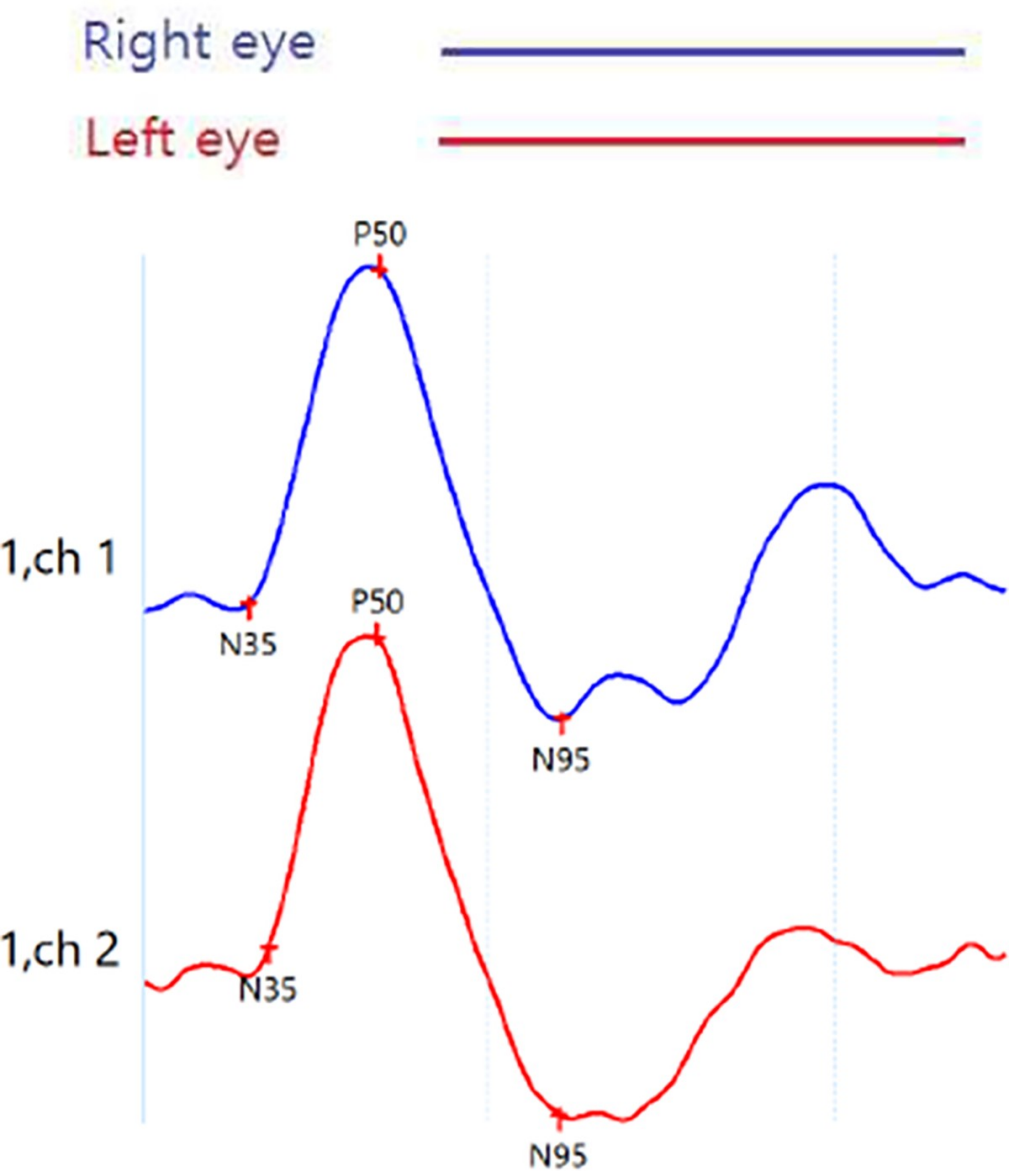
Representative waveform recording from PERG examination.

The amplitude and implicit time of P50 and N95 were measured. P50 amplitude was determined as the height from the trough of N35 to the peak of P50. Amplitude of N95 was measured from the P50 peak to the N95 trough. The representative waveform recording result was presented in Fig 1.

### Confocal scanning laser ophthalmoscopy (CSLO) of the optic disc

CSLO tomography of the optic disc was performed using the Heidelberg Retina Tomograph Ⅲ (HRT Ⅲ; Heidelberg Engineering, Heidelberg, Germany). HRT provides a way to acquire a 3D terrain image of the optic disc and objectively reconstruct the parameters of the optic disc morphology. Topographic images of disc were obtained through dilated pupils with size more than 6 mm, and each image was automatically corrected for retinal tilt. The margin of the optic disc was manually assigned on the image as a contour line around the inner edge of the Elschnig’s scleral ring by one trained technician. All images included in our analysis had passed the HRT-III's automatic scan quality checks, and the standard reference plane provided by the instrument was used. These parameters of optic disc and RNFL were recorded: linear cup/disc ratio (LCDR), cup shape measure (CSM), rim area, rim volume, height variation contour (HVC), mean RNFL thickness, and the linear discriminant function containing glaucoma probability score.[15–19]

### Statistical analysis

All statistical analyses were performed with SPSS version 24.0 (SPSS Inc., Chicago, IL, USA); *P* < 0.05 was considered to be statistically significant. Student t test and chi-square test were used to compare the characteristics and results of OCT, perimetry, and ERG between glaucoma suspect and NTG. Intraclass correlation coefficients (ICCs) were calculated for estimating variability in the electrophysiologic test results using data from 20 normal eyes tested twice. We used Pearson correlation analysis to evaluate the relationships between RNFL thickness and visual functional parameters such as ERG and perimetry and to calculate the correlation coefficients between HRT and ERG parameters by grouping subjects into glaucoma suspect and NTG. The linear and logarithmic R squares were calculated in scatter plots of RNFL thickness and ERG amplitudes or MD of perimetry.

## Results

A total of 86 eyes of glaucoma suspect subjects and 145 eyes of NTG patients were included in this study. Table 1 shows comparisons of the characteristics and demographic features in both groups; mean age and male to female ratios did not differ statistically. As predicted, average RNFL thickness, average cup-disc ratio, MD, and PSD of perimetry were all worse in NTG patients than in glaucoma suspect (all *P* < 0.001). In terms of PERG results, the latencies of P50 and N95 were elongated (*P* = 0.022 and 0.027), and amplitudes were lower in NTG (all *P* < 0.001). Table 2 presents the ICCs for latencies and amplitudes of PERG, and amplitudes of both P50 and N95 showed good to excellent repeatability (0.772 for P50 and 0.989 for N95).

**Table 1:** Characteristics of glaucoma suspect and normal tension glaucoma subjects.

	Glaucoma suspect (n = 86)	Normal tension glaucoma (n = 145)	*P* value
Age, y	54.34 (±12.78)	56.32 (±13.36)	0.270
Male:female	30:56	61:84	0.366
Average RNFL thickness, μm	90.83 (±6.93)	70.17 (±11.64)	<0.001
Average cup disc ratio	0.66 (±6.93)	0.75 (±6.93)	<0.001
**SITA 24–2**			
MD, dB	-0.83 (±1.61)	-7.04 (±7.25)	<0.001
PSD, dB	1.65 (±0.49)	5.88 (±4.40)	<0.001
**FDT 24–2**			
MD, dB	-4.26 (±3.03)	-9.48 (±6.04)	<0.001
PSD, dB	3.08 (±1.25)	5.15 (±2.38)	<0.001
**Pattern ERG**			
P50 latency, ms	49.53 (±3.15)	50.98 (±6.37)	0.022
N95 latency, ms	98.16 (±5.88)	100.93 (±10.61)	0.027
P50 amplitude, μV	3.14 (±0.86)	2.47 (±0.93)	<0.001
N95 amplitude, μV	5.79 (±1.34)	4.28 (±1.67)	<0.001

The ratios between males and females were compared using chi-square test. The others were compared using Student’s t test.

**Table 2:** First and second mean values and intraclass correlation coefficients (ICCs) of each parameter in pattern electroretinogram.

	Mean value at first time	Mean value at second time	ICC	*P* value
P50 latency, ms	50.15 (±3.47)	50.40 (±3.26)	0.378	0.222
N95 latency, ms	100.05 (±6.33)	101.01 (±4.44)	0.101	0.431
P50 amplitude, μV	4.52 (±1.03)	4.30 (±0.66)	0.772	0.011
N95 amplitude, μV	7.63 (±1.66)	7.51 (±1.51)	0.989	<0.001

We measured the correlations between average RNFL thickness and the functional parameters SITA 24–2, FDT 24–2, and PERG (Tables 3 and 4). In all subjects and NTG patients, there were significant correlations in amplitudes of P50 and N95, MD of SITA 24–2, and FDT 24–2 with average RNFL thickness. However, in the glaucoma suspect group, MD of both SITA and FDT did not show significant correlations with RNFL thickness; only N95 amplitude of PERG presented significant correlations (*P* = 0.024). The correlations between average GCIPL thickness and the functional parameters were also evaluated, and the results showed similar pattern except the point that only P50 amplitude presented significant correlations (*P* = 0.029).

**Table 3:** Correlation coefficients for RNFL thickness and GCIPL thickness with pattern ERG and perimetry in total subjects.

	RNFL thickness		GCIPL thickness	
	r	*P* value	r	*P* value
P50 latency	-0.043	0.519	0.068	0.371
N95 latency	-0.091	0.170	-0.002	0.977
P50 amplitude	0.368	<0.001	0.386	<0.001
N95 amplitude	0.533	<0.001	0.557	<0.001
SITA 24–2 MD	0.645	<0.001	0.586	<0.001
FDT 24–2 MD	0.639	<0.001	0.597	<0.001

Pearson correlation analysis was used.

**Table 4:** Correlation coefficients for RNFL thickness and GCIPL thickness with pattern ERG and perimetry after grouping glaucoma suspect and normal tension glaucoma.

	RNFL thickness					GCIPL thickness			
	Glaucoma suspect		Normal tension glaucoma			Glaucoma suspect		Normal tension glaucoma	
	r	*P* value	r		*P* value	R	*P* value	R	*P* value
P50 latency	-0.148	0.175	0.106	0.204		0.001	0.995	0.098	0.297
N95 latency	0.014	0.901	0.017	0.836		0.260	0.054	0.031	0.742
P50 amplitude	0.150	0.172	0.216	0.009		0.285	0.029	0.279	0.003
N95 amplitude	0.243	0.024	0.401	<0.001		0.176	0.177	0.506	<0.001
SITA 24–2 MD	0.129	0.269	0.557	<0.001		0.154	0.291	0.505	<0.001
FDT 24–2 MD	0.096	0.514	0.586	<0.001		0.194	0.212	0.486	<0.001

Pearson correlation analysis was used.

**Fig 2:**
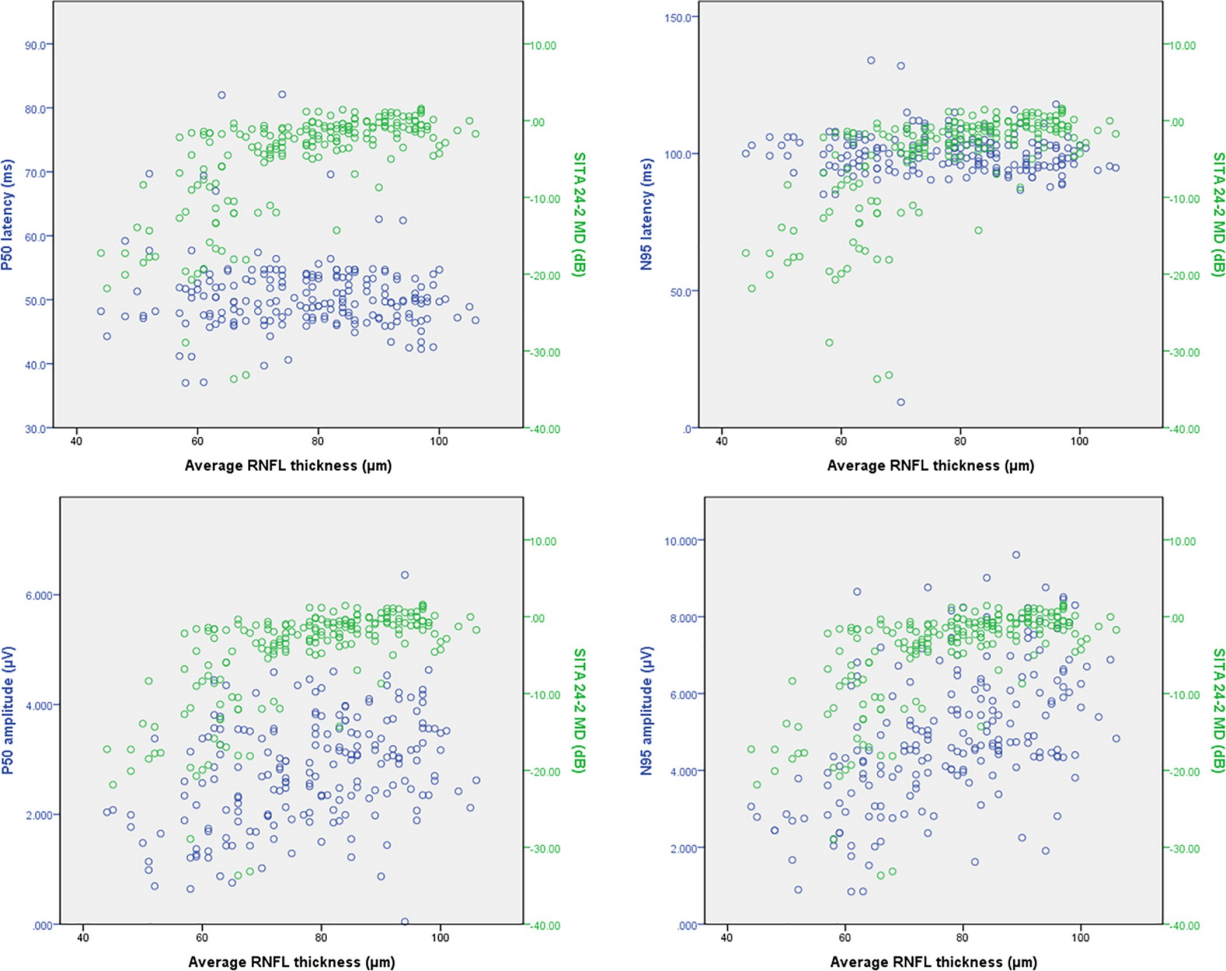
Scatter plot showing parameters of pattern ERG and SITA 24–2 MD by average RNFL thickness.

**Fig 3:**
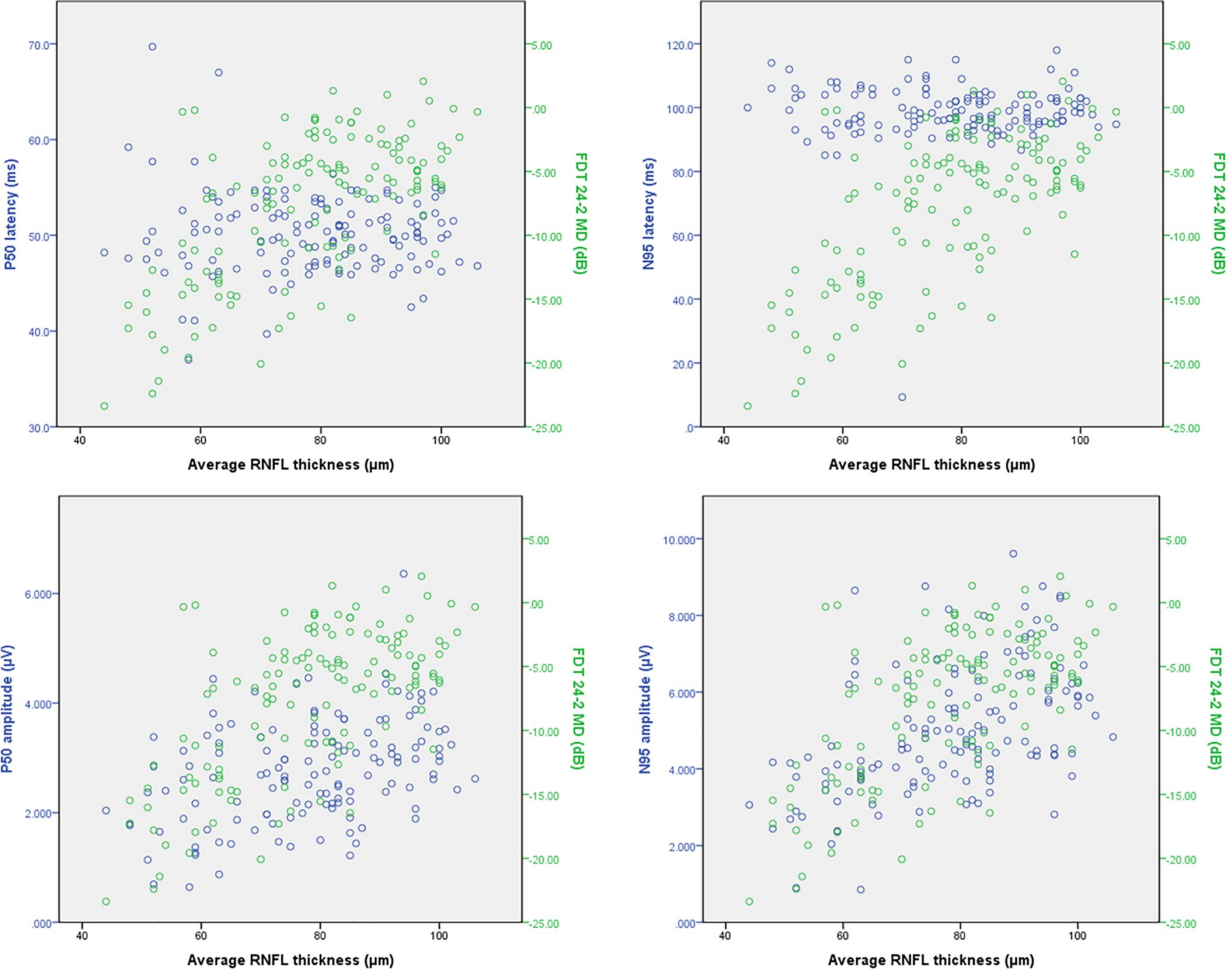
Scatter plot showing parameters of pattern ERG and FDT 24–2 MD by average RNFL thickness.

Scatter plots between RNFL thickness and visual functional parameters showed different patterns depending on the type of test. For Fig 2 we compared the scatter plots of PERG and SITA 24–2; the MD of SITA 24–2 was relatively consistent in the thick RNFL range but decreased dramatically when MD began to decrease. Fig 3 shows the scatter plots of PERG and FDT 24–2 for the same graphs, and the MD of FDT showed a more similar relationship with PERG amplitudes than with SITA.

**Fig 4:**
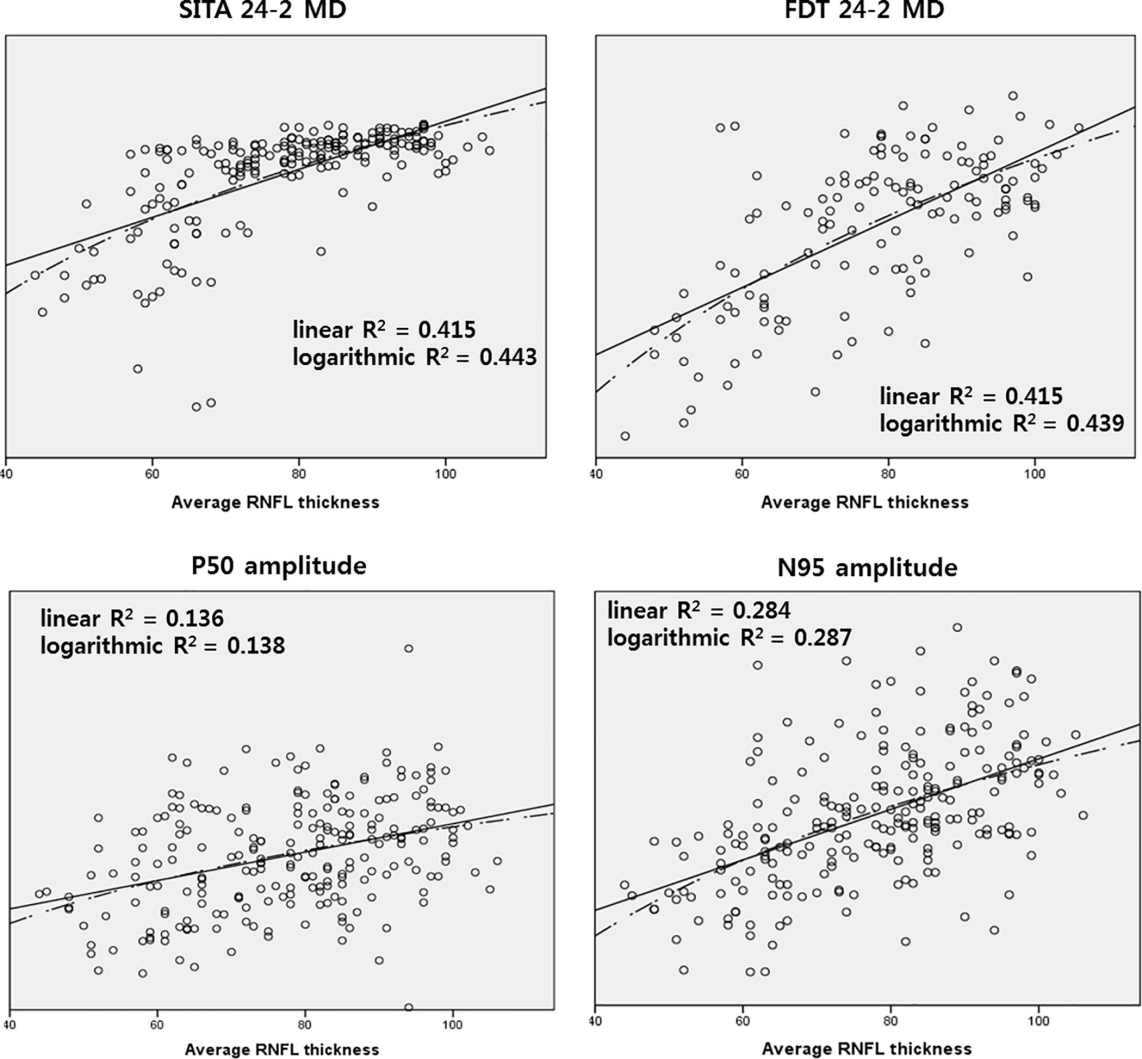
Linear and logarithmic R square values between average RNFL thickness and functional parameters (pattern ERG amplitude, MD of perimetry).

Fig 4 shows the comparisons of linear and logarithmic R^2^ in all visual functional parameters with RNFL thickness. The difference between linear and logarithmic R^2^ was largest for MD of SITA 24–2 (R^2^ for linear regression line = 0.415 and logarithmic regression line = 0.443), followed by order of FDT 24–2 MD, N95 amplitude, and P50 amplitude. In other words, to evaluate RNFL thickness, PERG amplitudes showed more linear relationships than other perimetries.

**Fig 5:**
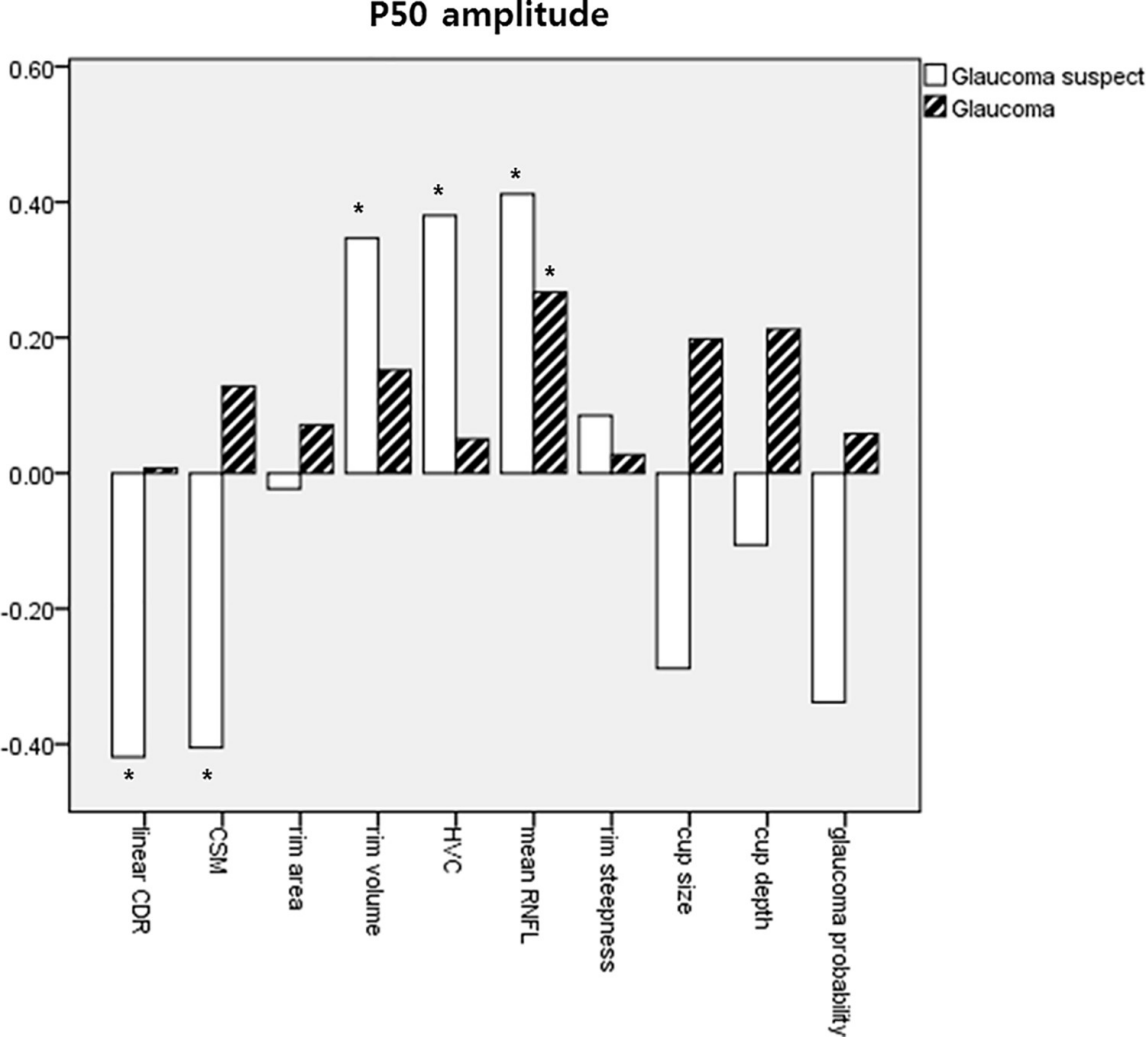
Correlation coefficients between P50 amplitude and HRT parameters. * *P* value less than 0.05.

**Fig 6:**
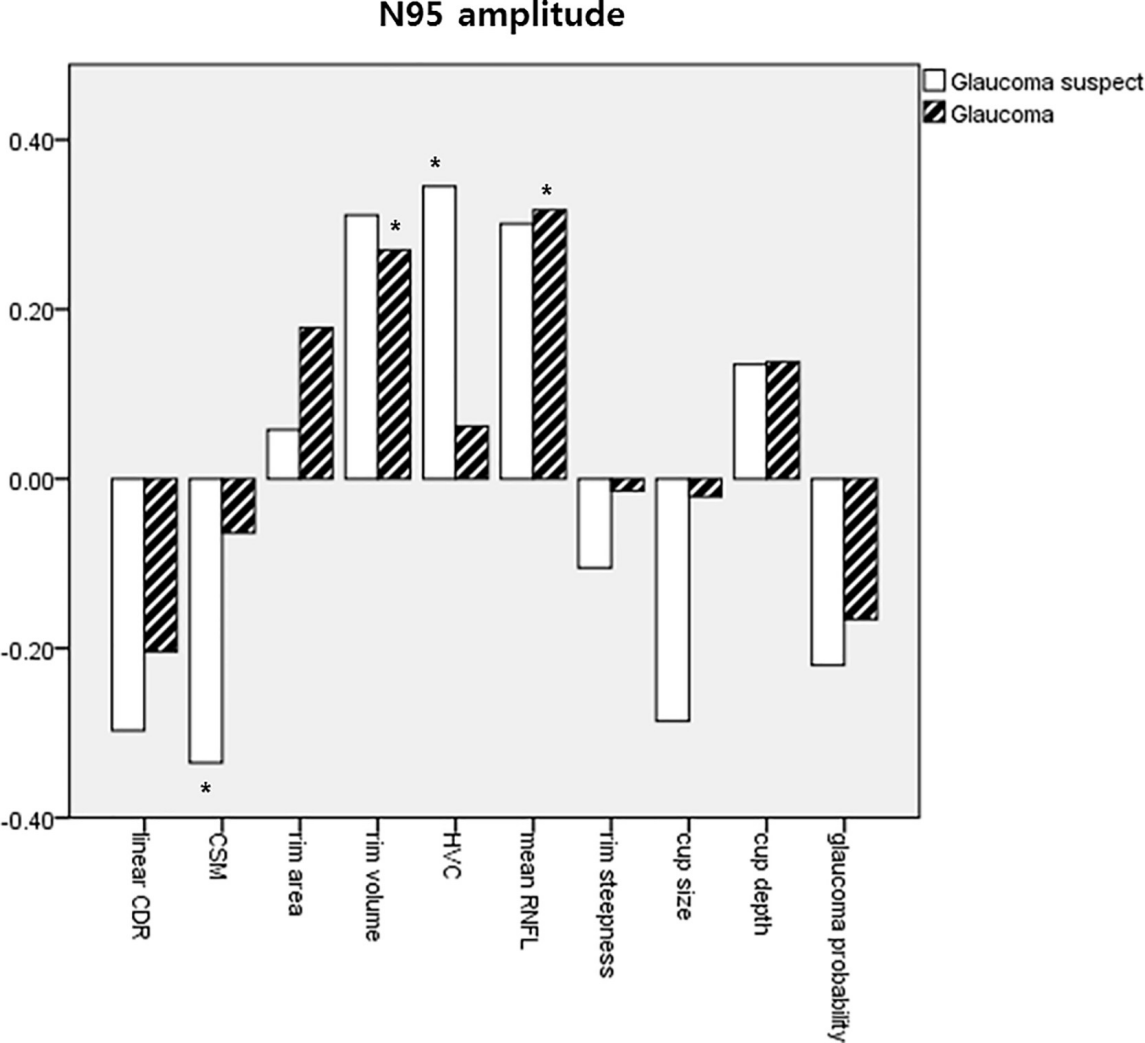
Correlation coefficients between N95 amplitude and HRT parameters. * *P* value less than 0.05.

Tables 5 and 6 show the results of our comparisons of disc morphological parameters of HRT with PERG amplitude. In glaucoma suspect, CSM and HVC correlated significantly with both P50 and N95 amplitudes (*P* = 0.016 and 0.049 for CSM; 0.024 and 0.042 for HVC) and was correlated with P50 amplitude (*P* = 0.012). However, CSM, HVC, and LCDR did not show significant correlations with PERG amplitudes in NTG patients. Rather, mean RNFL was correlated with P50 and N95 amplitudes (*P* = 0.011 and 0.002), and rim volume showed a significant correlation coefficient with N95 amplitude (*P* = 0.010). The correlation coefficients between HRT parameters and PERG amplitudes were summarized as graphs in Figs 5 and 6 grouping subjects as glaucoma suspect or glaucoma patients.

**Table 5:** Correlation coefficients for HRT parameters with pattern ERG amplitude in glaucoma suspect subjects.

	P50 amplitude		N95 amplitude		SITA 24–2 MD		FDT 24–2 MD	
	r	*P* value	r	*P* value	r	*P* value	r	*P* value
Linear CDR	-0.419	0.012*	-0.297	0.083	-0.029	0.894	0.162	0.472
CSM	-0.405	0.016*	-0.335	0.049*	-0.041	0.847	0.138	0.540
Rim area	-0.023	0.898	0.058	0.741	-0.101	0.637	0.134	0.551
Rim volume	0.347	0.041*	0.311	0.069	0.282	0.182	0.119	0.598
HVC	0.380	0.024*	0.345	0.042*	0.235	0.269	0.103	0.648
Mean RNFL	0.412	0.014*	0.301	0.079	0.338	0.106	-0.027	0.907
Rim steepness	0.085	0.626	-0.105	0.546	-0.467	0.021*	-0.065	0.772
Cup size	-0.288	0.093	-0.286	0.096	0.075	0.727	0.074	0.743
Cup depth	-0.106	0.546	0.135	0.439	0.436	0.033*	-0.071	0.754
Glaucoma probability	-0.338	0.047*	-0.220	0.204	0.007	0.974	-0.015	0.947

Pearson correlation analysis was used.

* *P* value less than 0.05

**Table 6:** Correlation coefficients for HRT parameters with pattern ERG amplitude in normal tension glaucoma subjects.

	P50 amplitude		N95 amplitude		SITA 24–2 MD		FDT 24–2 MD	
	r	*P* value	r	*P* value	r	*P* value	r	*P* value
Linear CDR	0.007	0.947	-0.204	0.053	-0.359	0.003*	-0.406	0.001*
CSM	0.128	0.228	-0.064	0.552	-0.191	0.125	-0.337	0.009*
Rim area	0.071	0.504	0.178	0.094	0.299	0.015	0.356	0.006*
Rim volume	0.152	0.152	0.270	0.010*	0.347	0.004*	0.347	0.007*
HVC	0.050	0.638	0.062	0.561	0.005	0.969	-0.073	0.584
Mean RNFL	0.267	0.011*	0.317	0.002*	0.452	<0.001*	0.355	0.006*
Rim steepness	0.027	0.809	-0.014	0.901	0.248	0.054	0.334	0.014*
Cup size	0.198	0.072	-0.021	0.849	-0.047	0.719	-0.072	0.605
Cup depth	0.212	0.054	0.138	0.214	-0.052	0.689	-0.031	0.826
Glaucoma probability	0.058	0.604	-0.166	0.133	-0.263	0.041*	-0.254	0.064

Pearson correlation analysis was used.

* *P* value less than 0.05

## Discussion

This study showed the correlations between PERG amplitude with RNFL thickness and disc morphology even in glaucoma suspect without IOP elevation. Glaucoma suspect subjects with only enlarged C/D ratio (without high IOP or other glaucomatous disc change) were included in our study, and in our view, we are the first to report on evaluating the meaning of PERG along with disc morphology in those subjects.

Glaucomatous optic neuropathy initiates as mechanical stress in the optic disc, especially in the lamina cribrosa, and it progresses as nerve fiber layer thinning and visual field loss.[20,21] Thus, there must be a stage that lies between only receiving stress in the lamina cribrosa and obvious appearance of retinal neuron loss. The stage is different from preperimetric glaucoma, because preperimetric glaucoma refers to definite neuronal loss without perimetric deterioration. In clinical practice, glaucoma suspect with large C/D ratio was considered functionally normal, but doubt remains about the influence of morphologic changes to the optic disc.

Visual field tests such as standard automated perimetry using SITA 24–2 program or FDT are common for evaluating visual functional status in glaucoma. However, VF tests are perceptional, based on patients’ subjective responses, so multiple individual factors including visual acuity, cognition, and physical restriction could affect the results. In addition, due to the substantial proportion of RGC damage prior to the occurrence of definite VF defect, there could be delay in detecting visual functional problems using only a VF test.[22,23] As expected, in the scatter plots of our results, MD of SITA showed the most curvilinear trend, in plateau state for thick RNFL but rapidly decreasing with RNFL thinning. MD of FDT showed a less curvilinear relationship than did SAP but less of a linear trend than amplitudes of PERG. FDT was usually accepted as an early diagnostic tool in glaucoma exceeding SAP,[24–26] but there still might be timing discrepancy in detecting the structural loss of RGC.

Electrophysiological tests such as electroretinogram directly record the response of the inner retina and may supplement the weaknesses of perimetry. It has been reported that PERG selectively represents the RGC function,[13] and each waveform means different origin–P50 from RGC soma and N95 from RGC axon.[27] Arden et al. asserted that PERG is generated from inner retina signaling,[28] and other study authors suggested earlier changes in PERG parameters than structural thinning of the retinal neuron.[1,29–31] In similar manners, in this study, the factor that had significant correlation with RNFL thickness in glaucoma suspect was only N95 amplitude of PERG. In the scatter plots of RNFL thickness and ERG amplitude, there were the earliest decreases according to RNFL thickness changes.

The interesting points in this study were the significant correlations between amplitude of ERG and parameters representing cup morphology in the glaucoma suspect group. Linear CDR and CSM showed meaningful relationships with PERG amplitudes, which described the contour of the optic cup. Changes of the optic disc precede RNFL thinning in glaucoma.[32] Over the course of morphologic changes of the cup, there were RGCs with various function despite the normal RNFL thickness range. After patients had progressed toward glaucomatous damage, ERG amplitudes showed significant correlations with mean RNFL but not with HVC or CSM, which were meaningful factors in glaucoma suspect. Subsequent to a certain period of lamina cribrosa change inside the cup, that is, when the actual decrease of neurons starts to take place, the degree of RNFL thinning and ganglion cell function become more related.

Based on the above findings, we could assume that the morphological changes of the optic disc occur first, followed by the ganglion cell dysfunction, and that thereafter, the sustained mechanical stress may be expected to cause physical reduction of the nerve axon. In other words, the presence of dysfunctional RGC can be confirmed by PERG in glaucoma suspect with intact neuronal structure. The functions of these RGCs could be affected by how much the cup has changed in the lamina cribrosa.

There could be some questions about re-use of electrophysiological test that was used in the past but was not so attractive under clinical situation. The equipment used in this study can test both eyes simultaneously and quickly without the difficulty of corneal lens applying, and it might reduce the variability caused by subjects’ variable reactions. It is valuable that this time-saving measurement could estimate the existence of dysfunctional RGC without RNFL loss in glaucoma suspect. However, this study has the intrinsic limitation of a cross-sectional design, and as such, it is difficult to predict the progression probability or prognosis of study subjects. In the future study, a longitudinal prospective study that could assess serial change of structure and functional parameters should be performed for clinical application of electrophysiologic test. In addition, this study excluded patients with increased IOP and it could make the results of this study not including all type of glaucoma.

In conclusion, RGC dysfunction could occur before structural loss of the retinal neuron based on cup contour variation. The functional changes to the retinal neuron may take place continuously during the period of mechanical stress that enlarges the C/D ratio but not sufficiently to thin the nerve layer. In a future study, the concept of functional RGC measurement using PERG could be applied in order to assess the effects of neuroprotective strategies for identifying RGC function restoration in dysfunctional but reversible ganglion cells with intact structure.

## Supporting information

S1 File Annonymized data. The annonymized data sheet from study subjects.
(XLSX)

S2 File Statistical results data. The statistical results data from study subjects.
(ZIP)
